# Constructing Cu_3_P Quantum Dots/Cu-Doped ZnIn_2_S_4_ p-n Heterojunctions for Efficient Methanol Oxidation Coupled with Synchronous Hydrogen Generation

**DOI:** 10.3390/nano16030210

**Published:** 2026-02-06

**Authors:** Maobin Xiao, Ke Wang, Jinghang Xu, Jie Hu, Weikang Wang, Lele Wang, Qinqin Liu

**Affiliations:** School of Materials Science & Engineering, Jiangsu University, Zhenjiang 212013, China

**Keywords:** photocatalysis, methanol activation, ethylene glycol, H_2_ generation, formaldehyde

## Abstract

The solar-driven direct conversion of methanol to ethylene glycol, formaldehyde and simultaneous H_2_ generation is an appealing strategy for converting sunlight to chemical energy. However, the low efficiency and stability of the photocatalyst remain critical bottlenecks hindering the practical implementation of this reaction. Herein, we synthesized the Cu_3_P quantum dots/Cu-doped ZnIn_2_S_4_ p-n junction for efficient methanol oxidation and synchronous H_2_ generation. The highly dispersed Cu_3_P quantum dots promote electron–hole separation and furnish abundant catalytic sites. Moreover, the constructed p-n junction with a tight interface boosts the electron transfer, avoiding the serious photocorrosion of ZnIn_2_S_4_. Benefiting from these synergistic effects, the 2Cu_3_P/Cu_0.5_ZIS composite exhibits the highest photocatalytic conversion efficiency of methanol, yielding H_2_, formaldehyde, and ethylene glycol with 10.34 mmol·g^−1^·h^−1^, 10.35 mmol·g^−1^·h^−1^ and 8.84 mmol·g^−1^·h^−1^ yields, which are 3.01, 3.05 and 3.10 times those of pure ZnIn_2_S_4_, respectively. A series of characterizations including X-ray diffraction, X-ray photoelectron spectroscopy, transmission electron microscopy and UV-Vis diffuse reflectance spectroscopy are employed to analyze the structure, composition, and photoelectrochemical properties of the materials. This work demonstrates a novel catalyst design paradigm for the high-efficiency solar light-driven photocatalytic activation of methanol enabling the co-production of value-added C1/C2 oxygenates and clean H_2_ fuel simultaneously.

## 1. Introduction

The surging global demand for solar-powered clean energy and high-value chemicals has spurred intensive research aimed at addressing the pressing challenges of environmental sustainability and energy utilization [1,2,3,4,5,6,7,8]. As a versatile renewable C1 building block and an appealing hydrogen (H_2_) carrier, methanol can be transferred into high-value products (ethylene glycol and formaldehyde) and H_2_ energy via redox reactions [9,10,11]. However, existing strategies for the conversion of methanol to organic products (ethylene glycol and formaldehyde) and H_2_ always show limited activity and poor catalyst stability [12,13]. Thus, there is an urgent need to design highly efficient and stable photocatalysts for simultaneous oxidation and reduction of alcohols.

Sulfide semiconductors have emerged as highly promising materials in different fields such as photocatalysis, photoelectrocatalysis, gas sensing, environmental remediation, etc., owing to their favorable band structures, excellent light absorption capability, and tunable morphologies [14,15,16,17,18]. Among these, ZnIn_2_S_4_ is a crucial ternary chalcogenide semiconductor, which has shown potential in the activation of alcohol molecules [19,20]. Like most pure-phase semiconductors, ZnIn_2_S_4_ suffers from high exciton binding energy and low carrier mobility, which lead to low reactivity, while severe photocorrosion restricted its practical application [21,22]. To overcome these limitations, a series of strategies including doping, heterojunction construction and co-catalyst introduction have been put forward [23,24,25]. Recently, Li et al. designed a Ni-doped ZnIn_2_S_4_ for improving the photocatalytic performance of aromatic alcohol dehydrogenative coupling and synchronous H_2_ evolution via enhancing the light absorption and charge carrier separation [26]. Li et al. fabricated a 1T/2H MoSe_2_/ZnIn_2_S_4_ S-scheme heterojunction that enhances structural stability and charge separation, enabling efficient photocatalytic hydrogen evolution coupled with benzyl alcohol oxidation [27]. All these pioneering works indicate that doping engineering and heterojunction construction serve as the feasible approaches to enhance the performance of ZnIn_2_S_4_. Currently, most research efforts focus on a single strategy to enhance catalyst performance, and there are scarce reports on the simultaneous adoption of both approaches to address the issues of inferior catalytic activity and poor stability [28,29]. Indeed, constructing heterojunctions is also a viable method for achieving internal modification. The incorporation of copper atoms can effectively broaden the light absorption of the semiconductors and create more active sites (such as vacancies or metal-doped sites), thereby significantly enhancing its photocatalytic performance [30,31,32]. In addition, Cu_3_P quantum dots (QDs) as a p-type semiconductor can couple with the Cu_x_ZnIn_2_S_4_ which improves the redox ability and stability through promoting the charge transfer.

Herein, we constructed a p-n heterojunction composed of Cu-doped ZnIn_2_S_4_ nanosheet and Cu_3_P QDs for efficient methanol oxidation to ethylene glycol and formaldehyde and synchronous reduction to H_2_. As a result, the optimized 2Cu_3_P/Cu_0.5_ZIS composite achieved high yields of H_2_, formaldehyde, and ethylene glycol of 10.34 mmol·g^−1^·h^−1^, 10.35 mmol·g^−1^·h^−1^ and 8.84 mmol·g^−1^·h^−1^, which are 3.01, 3.05 and 3.10 times those of pure ZnIn_2_S_4_, respectively. The enhanced activity and stability may be due to the synergistic effect of Cu doping and p-n heterojunction construction, which not only promote the charge separation but also afford abundant spatially separated active sites. The Cu doping broadened the light absorption and created more vacancies. The tight interfacial contact not only improved the charge separation, thereby suppressing the photocorrosion of ZnIn_2_S_4_, but also provides oxidation reaction sites for methanol molecules, enhances charge transfer efficiency, and increases the surface electron concentration. This work demonstrates an effective strategy for constructing heterostructure photocatalysts with efficient photocatalytic methanol oxidation and synchronous H_2_ generation.

## 2. Experimental Section

### 2.1. Preparation of Cu_3_P QDs

The Cu_3_P QDs were synthesized via a calcination method. As is typical, 1 g CuCl_2_·H_2_O and 5 g NaH_2_PO_2_·H_2_O were mixed in a flask with continuous grinding for half an hour. Then, the mixture was transferred to a porcelain boat and kept at 300 °C in N_2_ atmosphere for 2 h. The crude product was then centrifuged and washed three times with deionized water. After vacuum drying at 60 °C for 24 h, the gray powder Cu_3_P quantum dots were obtained.

### 2.2. Preparation of ZnIn_2_S_4_ (ZIS) and Cu_x_ZnIn_2_S_4_ Nanosheets (Cu_x_ZIS, x = 0.3, 0.5 and 0.7)

The ZIS and Cu_x_ZIS nanosheets were synthesized according to our previously reported method. For the preparation of ZIS nanosheet, 175.6 mg Zn(Ac)_2_·2H_2_O, 469.1 mg InCl_3_·4H_2_O and 150 mg TAA were dissolved in 60 mL of a mixed solvent including deionized water and ethanol (volume ratio = 1:1) with stirring for 30 min. Then, the above solution was moved to a stainless steel autoclave and kept at 180 °C for 24 h. Upon cooling to 25 °C, the production was washed three times with ethanol and deionized water. Finally, ZIS was obtained by freeze-drying at 60 °C overnight. Similarly, Cu_x_ZIS nanosheets were also prepared by the above procedure, except that 0.3, 0.5 and 0.7 mL 0.1 M Cu(NO_3_)_2_ were added to the solution before the hydrothermal process, respectively.

### 2.3. Synthesis of Cu_3_P/Cu_x_ZIS Heterojunction

The Cu_3_P/Cu_x_ZIS compositions were synthesized via an electrostatic self-assembly process. Typically, 100 mg Cu_x_ZIS ultrathin nanosheets was added to 20 mL DMF with ultrasonic vibration for 30 min, adding a certain amount of Cu_3_P quantum dots (0.05, 0.10 and 0.15 mg·mL^−1^) and 20 mL ethanol into the above Cu_x_ZIS aqueous. After sonicating for 20 min, the resulting sample was washed twice with ethanol, and then dispersed in a 50 mL ethanol solution with stirring for 8 h. Conclusively, the samples were subjected to three ethanol washes and subsequent drying under a vacuum at 60 °C, yielding 1Cu_3_P/Cu_0.5_ZIS, 2Cu_3_P/Cu_0.5_ZIS, and 3Cu_3_P/Cu_0.5_ZIS, respectively.

### 2.4. Photocatalytic Activity

The photocatalytic reaction was carried out in a 20 mL sealed quartz-tube reactor under a 300 W Xe lamp. The solid catalyst powder (10 mg) was dispersed in 3 mL of mixture containing 2.5 mL methanol and 0.5 mL H_2_O. Before light irradiation, the reactor was evacuated by vacuum pump. Then, the photocatalytic reaction was carried out by continuous irradiation of the reactor with a full-spectrum Xe lamp for 6 h at 25 °C. After the reaction, the gaseous products were extracted and analyzed by an online gas chromatograph (Tian Mei GC-7900, argon as carrier gas), and the supernatant of the solution was extracted and analyzed by gas chromatography.

The detailed information for materials and material characterization can be seen in the supporting information.

## 3. Results and Discussion

**Figure 1:**
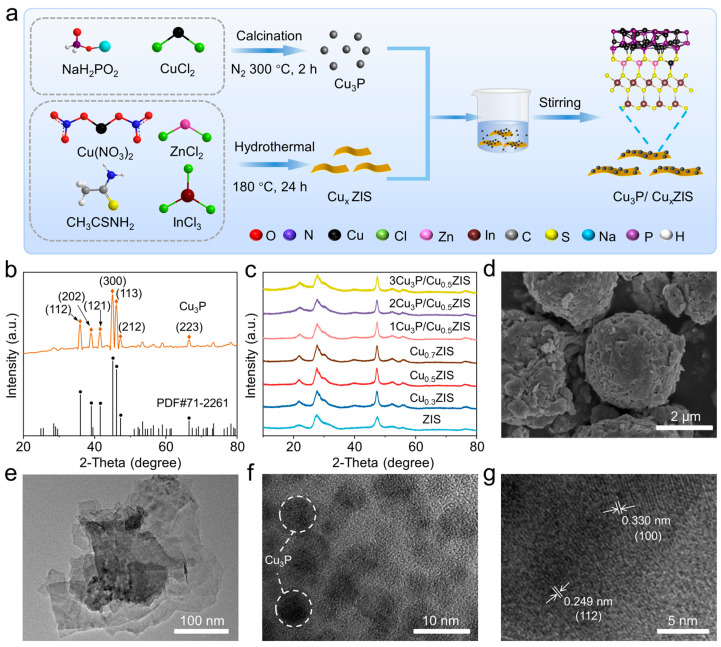
(**a**) Synthesis of Cu_3_P/Cu_x_ZIS heterostructure. (**b**,**c**) XRD patterns of different samples. (**d**) SEM image of ZIS samples. (**e**–**g**) HRTEM images of Cu_0.5_ZIS, Cu_3_P and 2Cu_3_P/Cu_0.5_ZIS samples.

The synthesis of the 0D/2D Cu_3_P quantum dot CuxZIS nanosheet composites is illustrated in Figure 1a. The crystal structure and microstructures of the samples were clearly verified by X-ray diffraction (XRD) patterns, scanning electron microscopy (SEM) and transmission electron microscopy (TEM) measurements [33,34,35]. The peaks centered at 36.04°, 39.12°, 41.60°, 45.17°, 46.20°, 47.35° and 66.58° can be indexed to the (112), (202), (121), (300), (113), (212) and (223) crystal planes of a hexagonal phase of Cu_3_P (PDF#71-2261), respectively (Figure 1b) [36]. Compared with pure ZIS, no change is observed in the XRD patterns of Cu_x_ZIS (x = 0.3, 0.5 and 0.7), indicating that trace Cu atom doping did not change the crystal phase (Figure 1c) [37]. As shown in Figure 1d, pure ZIS exhibited 3D flower spheres composed of nanosheets. Different from the pristine ZIS flower spheres, the Cu_x_ZIS presented an ultrathin nanosheet morphology with an average size of around 100 nm after Cu doping, which exposed more active sites for photoreactions (Figure 1e). Cu_3_P was observed as quantum dot structures with an average size of 5 nm (Figure 1f). A TEM image of 2Cu_3_P/Cu_x_ZIS heterojunction indicated that Cu_3_P quantum dots deposited on the surface of Cu_x_ZIS nanosheets. The lattice spacing of Cu_x_ZIS nanosheets was 0.330 nm, corresponding to the (110) phase of ZIS (Figure 1g). The lattice spacing of 0.249 nm on Cu_3_P corresponds to the (112) face. Partially distorted lattice fringes can be observed in Cu_x_ZIS nanosheets, which were induced by Cu doping. Meanwhile, the crystalline Cu_3_P phase was found to be regularly dispersed in the vicinity of the ZIS distorted lattice, which serves as direct evidence that Cu doping directionally induces Cu_3_P deposition (Figures S1 and S2). The energy-dispersive X-ray (EDX) element mapping and HRTEM images in Figure S3 showed that Zn, In, S, Cu and P elements were uniformly distributed in the sample, which proved the successful synthesis of the 2Cu_3_P/Cu_0.5_ZIS heterojunction.

**Figure 2:**
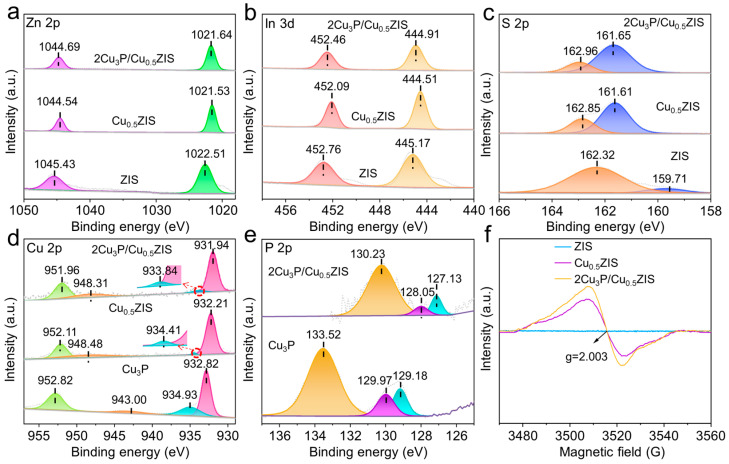
High-resolution XPS spectra of Zn 2p (**a**), In 3d (**b**), S 2p (**c**), Cu 2p (**d**) and P 2p (**e**). (**f**) EPR spectra for the S vacancy of ZIS, Cu_0.5_ZIS and 2Cu_3_P/Cu_0.5_ZIS.

X-ray photoelectron spectroscopy (XPS) was also tested to investigate the chemical composition of these samples [38,39]. The survey spectra show the presence of Zn, In, S, P and Cu elements in the 2Cu_3_P/Cu_0.5_ZIS composites, confirming the successful synthesis (Figure S4). The high-resolution Zn 2p XPS spectroscopy showed two peaks located at 1022.51 and 1045.43 eV, belonging to the binding energy of Zn 2p_3/2_ and Zn 2p_1/2_, respectively (Figure 2a) [40]. The In 3d spectra revealed two peaks at 445.17 and 452.76 eV corresponding to the 3d_3/2_ and 3d_1/2_ signals of In, respectively (Figure 2b) [41]. The S 2p peaks of ZIS showed two peaks at 159.71 and 162.32 eV, attributed to S 2p_3/2_ and S 2p_1/2_, respectively (Figure 2c) [40]. The Zn 2p and In 3d peaks in Cu_0.5_ZIS shifted to lower bonding energy, while the peaks of S 2p in the Cu_0.5_ZIS show a higher bonding energy compared with pure ZIS. The results indicate that Cu doping can lead to the generation of S vacancies (Vs), which induced a rearrangement of charges on the ZIS surface and might be favorable for the loading of Cu_3_P. The Cu 2p XPS spectra of Cu_3_P can be deconvoluted into four peaks at 932.82, 934.93, 943.00 and 952.82 eV, corresponding to Cu 2p_1/2_ and Cu 2p_3/2_, respectively (Figure 2d) [42]. Three peaks at 129.18, 129.97 and 133.52 eV in P 2p XPS spectra can be found, which are ascribed to the P 2p_3/2_, P 2p_1/2_ and P-O bond, respectively (Figure 2e) [42]. In the Cu_3_P/Cu_0.5_ZIS composites, the signals for Zn 2p, In 3d and S 2p shifted toward higher bonding energies while the Cu 2p and P 2p signals shifted to the lower bonding energies compared to Cu_0.5_ZIS and Cu_3_P, respectively. A built-in electric field (IEF) is formed at the 2Cu_3_P/Cu_0.5_ZIS heterojunction interface directing from Cu_0.5_ZIS to Cu_3_P. In particular, the bonding energy of the P 2p_3/2_ peaks in the composite moved from 129.18 to 127.13 eV compared with pure Cu_3_P, indicating that the P ions were captured by the electron traps (Vs) in Cu_0.5_ZIS. Electron paramagnetic resonance (EPR) measurement was also conducted to analyze the formation of S vacancy (Vs). In Figure 2f, a strong signal at g = 2.003 was detected in the presence of Cu_0.5_ZIS, Cu_3_P and 2Cu_3_P/Cu_0.5_ZIS heterojunction, which accounts for the signal of Vs. No signal can be detected in ZIS. The above results indicated that the composition with an IEF at the interface will enhance the charge separation, affording high redox activity in the photocatalytic reactions.

**Figure 3:**
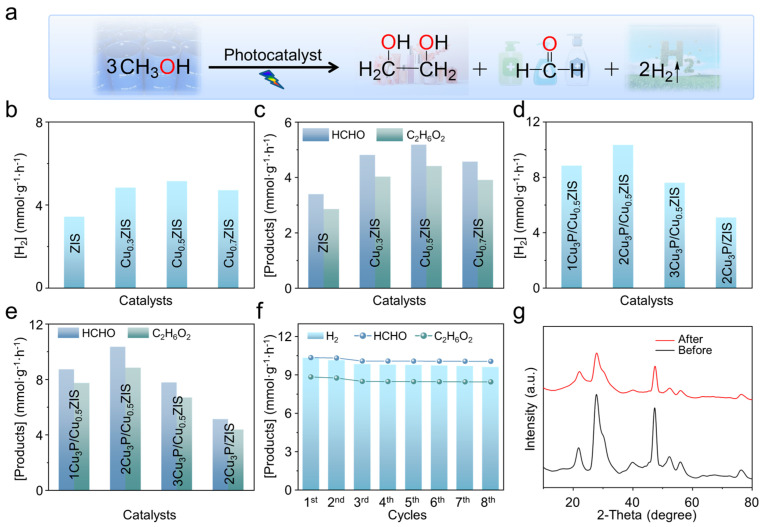
(**a**) Reaction equation for methanol activation. (**b**,**c**) The photocatalytic activity of different samples. (**d**,**e**) The photocatalytic activity of 1Cu_3_P/Cu_0.5_ZIS, 2Cu_3_P/Cu_0.5_ZIS, 3Cu_3_P/Cu_0.5_ZIS and Cu_3_P/ZIS samples. (**f**) The cyclic reaction of 2Cu_3_P/Cu_0.5_ZIS sample under light irradiation. (**g**) Recycled XRD pattern of 2Cu_3_P/Cu_0.5_ZIS sample.

To analyze the photocatalytic activity of these samples, the methanol dehydrogenation coupling H_2_ evolution reaction (HER) was carried out under UV-Vis-NIR light irradiation (Figure 3a). As illustrated in Figure 3b–e, all samples could simultaneously activate methanol to give H_2_, formaldehyde and ethylene glycol. Under light irradiation, methanol undergoes a redox reaction, in which the generated H^+^ was reduced by the electrons to generate H_2_ while the intermediate reacted with photogenerated holes to generate formaldehyde and ethylene glycol through an oxidation and C-C coupling reaction, respectively. Pure ZIS shows the H_2_ production of 3.43 mmol·g^−1^·h^−1^. After a reaction period of 6 h, the production of formaldehyde and ethylene glycol was measured at 3.39 mmol·g^−1^·h^−1^ and 2.85 mmol·g^−1^·h^−1^, respectively. The rate of H_2_, formaldehyde and ethylene glycol production improves with increasing Cu dopant. The Cu_0.5_ZIS shows the highest HER rate of 5.14 mmol·g^−1^·h^−1^, 5.18 mmol·g^−1^·h^−1^ for formaldehyde production and 4.41 mmol·g^−1^·h^−1^ for ethylene glycol production, which are 1.50, 1.52 and 1.54 times that of pure ZIS. However, the photocatalytic performance of the photocatalyst decreases with excess Cu doping. Furthermore, the introduction of Cu_3_P quantum dots further enhanced the catalytic activity of the Cu_0.5_ZIS. The highest H_2_, formaldehyde and ethylene glycol production can reach 10.34 mmol·g^−1^·h^−1^, 10.35 mmol·g^−1^·h^−1^ and 8.84 mmol·g^−1^·h^−1^ in the presence of 2Cu_3_P/Cu_0.5_ZIS photocatalyst, which is 3.01, 3.05 and 3.10 times that of pure ZIS, respectively. In addition, the photocatalytic performance for 2Cu_3_P/Cu_0.5_ZIS is higher than that of 2Cu_3_P/ZIS, proving that Cu doping-induced Cu_3_P loading can effectively improve the photocatalytic activity of ZIS to achieve far more than the direct loading of ZIS by Cu_3_P (Figure 3d,e). The calculated selectivities for formaldehyde and ethylene glycol are 53.93% and 46.07%, respectively, with a high methanol molar balance of 99% (Table S1 and Formulas (S1) and (S2)). The methanol conversion can reach 3.02% according to Formula (S3). As shown in Figure S5, no formaldehyde, ethylene glycol, or H_2_ can be detected under dark, catalyst-free and pure-solvent conditions. Adaptive vacancies generated via Cu doping enable the site-specific deposition of Cu_3_P quantum dots in the Vs, which efficiently suppresses electron–hole recombination and thus accounts for the enhanced catalytic activity. To further test the stability of the catalyst, the long-term photocatalytic activity of 2Cu_3_P/Cu_0.5_ZIS sample was also tested. As shown in Figure 3f, the production can be retained with no obvious decrease even after 8 rounds of irradiation. No significant change can be detected in the XRD and TEM patterns of the samples before and after the reaction, while a 0.08% mass loss can be observed, indicating the outstanding photo-stability (Figure 3g and Figure S6, Table S2). Compared with the previously reported works, the 2Cu_3_P/Cu_0.5_ZIS sample in this work exhibits higher photocatalytic H_2_ evolution, formaldehyde and ethylene glycol production (Table 1). All these results indicate that the Cu incorporation and coupling with Cu_3_P can significantly enhance the photocatalytic activity of ZIS.

**Figure 4:**
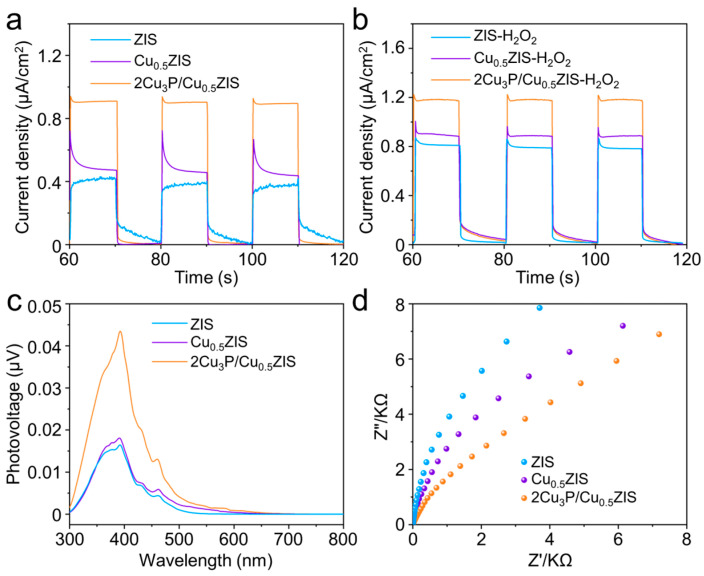
(**a**) Photocurrent response of the 2Cu_3_P/Cu_0.5_ZIS, Cu_0.5_ZIS and ZIS. (**b**) Photocurrent response of the 2Cu_3_P/Cu_0.5_ZIS, Cu_0.5_ZIS and ZIS with H_2_O_2_. (**c**) SS-SPV spectra of 2Cu_3_P/Cu_0.5_ZIS, Cu_0.5_ZIS and ZIS. (**d**) EIS Nyquist plots of 2Cu_3_P/Cu_0.5_ZIS, Cu_0.5_ZIS and ZIS.

To elucidate the reason for the improved photocatalytic activity of 2Cu_3_P/Cu_0.5_ZIS composite, the charge separation efficiency for the samples was investigated by photocurrent (PC) response, surface photovoltage (SPV), linear sweep voltammetry (LSV) and electrochemical impedance spectroscopy (EIS) measurement. As displayed in Figure 4a, the 2Cu_3_P/Cu_0.5_ZIS showed the highest photocurrent intensity, while the ZIS showed the lowest intensity, indicating that Cu doping and coupling with Cu_3_P can effectively enhance the carrier separation of the ZIS. Simultaneously, the surface charge transfer efficiency (η_trans_) was investigated by adding a fast electron scavenger (H_2_O_2_) to the electrolyte solution. As expected, the photocurrent intensities of all samples increase with the addition of H_2_O_2_ (Figure 4b). The photocurrent intensity of ZIS, Cu_0.5_ZIS and 2Cu_3_P/Cu_0.5_ZIS increases from 0.41, 0.48 and 0.88 μA·cm^−2^ to 0.80, 0.89 and 1.18 μA·cm^−2^, respectively. Accordingly, the η_trans_ values of ZIS, Cu_0.5_ZIS and 2Cu_3_P/Cu_0.5_ZIS samples were determined to be 51.3%, 54.0% and 74.5%, respectively, suggesting that the 2Cu_3_P/Cu_0.5_ZIS sample demonstrates faster carrier separation. The surface photovoltage (SPV) is also measured; compared with ZIS (0.016 μV) and Cu_0.5_ZIS (0.018 μV), a stronger photovoltage is observed in the 2Cu_3_P/Cu_0.5_ZIS sample, up to 0.043 μV (Figure 4c). The 2Cu_3_P/Cu_0.5_ZIS sample showed a lower overpotential than that of ZIS and Cu_0.5_ZIS, in the LSV curves, illustrating that the 2Cu_3_P/Cu_0.5_ZIS is more prone to HER (Figure S7). As shown in Figure 4d, the 2Cu_3_P/Cu_0.5_ZIS showed the smallest semicircle radius in EIS spectra, indicating that a Cu doping-induced Cu_3_P deposition strategy results in reduced interfacial charge transfer resistance between ZIS and Cu_3_P and accelerates carrier migration kinetics.

**Figure 5:**
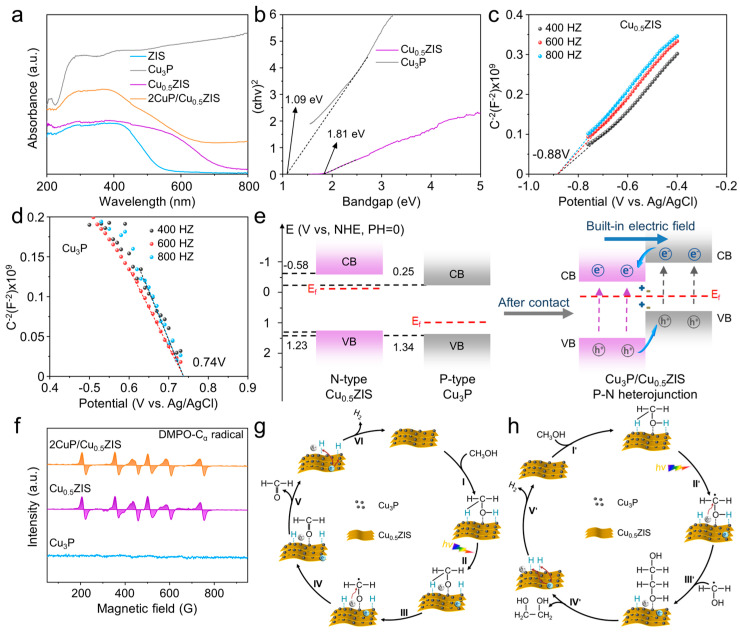
(**a**) DRS spectra of all samples. (**b**) The band structure of Cu_0.5_ZIS and Cu_3_P samples. Mott–Schottky plots of Cu_0.5_ZIS (**c**) and Cu_3_P (**d**) samples. (**e**) Band structure of Cu_0.5_ZIS and Cu_3_P samples. (**f**) EPR spectra of Cu_3_P, Cu_0.5_ZIS and 2Cu_3_P/Cu_0.5_ZIS samples. (**g**) Reaction mechanism of the generation of H_2_ and formaldehyde. (**h**) Reaction mechanism of the generation of H_2_ and ethylene glycol.

The light response and band structure of the samples were also tested. As shown in Figure 5a, the ZIS showed a wide light response range with an absorption edge at ~520 nm. After Cu doping, the adsorption edge of Cu_x_ZIS redshifted. The Cu_3_P quantum dots exhibit full-spectrum light-response ability. The loading of Cu_3_P quantum dots further enhances the light absorption of Cu_x_ZIS. The band gaps of ZIS and Cu_0.5_ZIS samples were calculated to be 2.43 and 1.81 eV via using the Kubelka–Munk function (detailed information can be seen in the supporting information), suggesting that Cu doping could narrow the band gap of ZIS nanosheets (Figure S8) [49,50,51]. The band gap of Cu_3_P is 1.09 eV (Figure 5b). The Mott–Schottky plots of Cu_0.5_ZIS and Cu_3_P processes show positive and negative slopes at different frequencies (at 400, 600, 800 Hz), revealing the n-type and p-type nature of the Cu_0.5_ZIS and Cu_3_P, respectively. Through the processing of these curves at three frequencies, the flat band potentials of Cu_0.5_ZIS and Cu_3_P were determined to be −0.88 V and 0.74 V, respectively (Figure 5c,d). The p-n type heterojunction was formed after interface contact between Cu_3_P and Cu_0.5_ZIS, inhibiting the recombination of electron–hole pairs (Figure 5e). When the Cu_3_P makes contact with Cu_0.5_ZIS, the electrons of Cu_0.5_ZIS spontaneously transfer to Cu_3_P at the interface of the heterojunction to equilibrate the Fermi energy (E_f_). The electrons migrate from Cu_0.5_ZIS to Cu_3_P, resulting in the formation of a built-in electric field directed from Cu_0.5_ZIS to Cu_3_P. Upon light excitation, the Cu3P and Cu0.5ZIS were excited. The photogenerated electrons transferred from the CB of Cu_3_P to Cu_0.5_ZIS while the holes transferred from the VB of Cu_0.5_ZIS to Cu_3_P, preventing their recombination via forming the p-n heterojunctions. These photogenerated holes and electrons take part in following surface redox reactions, thereby significantly enhancing the photocatalytic reaction activity.

Generally, there are two possible reaction pathways for the activation of methanol: one is to break the C-H bond to form a hydroxymethyl radical (·CH_2_OH, C_α_) and then generate ethylene glycol; another is to activate the O-H bond to form the formaldehyde via generating methoxy intermediate (CH_3_O*). To confirm the possible reaction pathways for methanol activation, EPR measurement and radical scavenger experiments were conducted. As shown in Figure 5f, when 5, 5-dimethyl-1-pyrroline-N-oxide (DMPO) was used as the trapping agent for the carbon radical intermediates, the signal corresponding to the C_α_ (·CH_2_OH) signal can be detected in the presence of 2Cu_3_P/Cu_0.5_ZIS heterojunction and Cu_0.5_ZIS. No signal can be seen with Cu_3_P as the photocatalyst. As shown in Figure S9, the addition of electron scavenger (nitrobenzene) reduced the H_2_ production rate rapidly, while the yields of formaldehyde and ethylene glycol remained unaffected. The addition of hole scavengers (Na_2_S/Na_2_SO_3_) led to a significant decrease in formaldehyde and ethylene glycol, accompanied by a slight increase in H_2_ evolution. The presence of a superoxide scavenger (1,4-benzoquinone, BQ) showed negligible effects on the generation of all the products. Furthermore, the addition of the ·CH_2_OH trapping agent 5,5-dimethyl-1-pyrroline N-oxide (DMPO) markedly suppressed the formation of both formaldehyde and ethylene glycol. These results confirm that the photogenerated carriers and ·CH_2_OH free radicals serve as the key species.

Based on these results, the reaction mechanism for photocatalytic methanol activation was proposed (Figure 5g,h). Before light excitation, the adsorption of methanol substrate molecules on the surface of 2Cu_3_P/Cu_0.5_ZIS catalyst takes place, which is a prerequisite step for the following reaction. Under light excitation, the generated electrons and holes accumulated on the surface of Cu_0.5_ZIS and Cu_3_P to take part in the redox reactions. Upon light irradiation, the 2Cu_3_P/Cu_0.5_ZIS was excited to generate electrons and holes. Driven by the functional difference between Cu_3_P and Cu_0.5_ZIS, the photogenerated electrons and holes accumulated on the CB and VB of Cu_0.5_ZIS and Cu_3_P, respectively [52,53]. The oxidation of C-H and O-H bond of methanol takes place on the surface of Cu_3_P, while the reduction of H^+^ to H_2_ takes place on the surface of Cu_0.5_ZIS. The α-C-H bond break of methanol results in the generation of ·CH_2_OH radical, affording ethylene glycol via subsequent C-C coupling. At the same time, the O-H bond can also be activated, forming the CH_3_O*. After α-C-H bond cleavage, formaldehyde can be obtained. The photogenerated electrons react with H^+^ stripped from methanol to produce H_2_.

## 4. Conclusions

In conclusion, a Cu_3_P/Cu_0.5_ZIS p-n heterojunction was constructed for the simultaneous production of H_2_ and value-added organics from methanol by coupling Cu_3_P quantum dots with Cu-doped Cu_x_ZIS ultrathin nanosheets. The heterojunction structure significantly enhances charge separation/migration, photocatalytic activity and stability. Using methanol as the substrate, C-H/O-H activation and H^+^ reaction were promoted to form ethylene glycol formaldehyde and H_2_ in a reaction system. The optimized 2% Cu3P/Cu0.5ZIS achieves H_2_ evolution of 10.34 mmol·g^−1^·h^−1^, with formaldehyde and ethylene glycol yields of 10.35 mmol·g^−1^·h^−1^ and 8.84 mmol·g^−1^·h^−1^, respectively, nearly 3.01-, 3.05- and 3.10-fold those of pure ZIS. Cu doping increases surface area and active sites, while Cu_3_P deposition establishes an efficient charge transfer pathway, improving carrier mobility, adsorption, light harvesting, and stability. This work provides an effective strategy for a highly active and stable semiconductor photocatalyst.

## Figures and Tables

**Table 1:** Comparison of the photocatalytic performance in methanol activation to generate H_2_ and oxidation products for different materials.

Photocatalyst	H_2_ (mmol·g^−1^·h^−1^)	Ethylene Glycol (mmol·g^−1^·h^−1^)	Formaldehyde (mmol·g^−1^·h^−1^)	References
2CuP/Cu_0.5_ZIS	10.34	8.84	10.35	This work
ZnIn_2_S_4_/TiO_2_-Cl	8.8	7.2	1.2	[43]
CdS	0.75	0.46	0.38	[44]
MoS_2_/CdS	12.0	11.0	2.5	[45]
0.1%-Ni-15%-ZIS/ZCS	11.2	12.5	trace	[11]
0.25CoP/Zn_2_In_2_S_5_	7.8	7.2	1.2	[46]
ZCS-Amine	6.2	5.1	1.0	[47]
BNH	0.49	0.46	trace	[12]
2%N-Ta_2_O_5_	8.6	4.0	2.5	[48]

## Data Availability

The original contributions presented in this study are included in the article/Supplementary Material. Further inquiries can be directed to the corresponding authors.

## Supplementary Materials

The following supporting information can be downloaded at https://www.mdpi.com/article/10.3390/nano16030210/s1.
