# Supplementary material for: Constructing Cu3P Quantum Dots/Cu-Doped ZnIn2S4 p-n Heterojunctions for Efficient Methanol Oxidation Coupled with Synchronous Hydrogen Generation
Source: Nanomaterials (Basel). 2026 Feb 6;16(3):210. doi: 10.3390/nano16030210 (PMC12899435; doi:10.3390/nano16030210)
Supplement: Supplementary file 1 [file nanomaterials-16-00210-s001.zip › nanomaterials-4110546-supplementary.pdf]

Supporting Information

Constructing Cu_3P Quantum Dots/Cu-Doped $ZnIn_2S_4$ p-n Heterojunctions to Realize Efficient Methanol Oxidation and Synchronous Hydrogen Generation

Maobin Xiao[a], Ke Wang[a], Jinghang Xu[a], Jie Hu[a], Weikang Wang[a], Lele Wang[a]* and Qinqin Liu[a]*

[a] *School of Materials Science & Engineering, Jiangsu University, Zhenjiang, Jiangsu 212013, P. R. China*

*Corresponding authors: e-mail: *llwang2020@ujs.edu.cn (L.W.)*, *qqliu@ujs.edu.cn (Q.L.)*

1. Materials

Copper chloride dihydrate ($CuCl_2 \cdot 2H_2O$, 98%), sodium hypophosphite monohydrate ($NaH_2PO_2 \cdot H_2O$, 98%), zinc acetate dihydrate ($Zn(Ac)_2 \cdot H_2O$, 98%), methanol (99%) and N,N-dimethylformamide (DMF, 99%) were obtained from Sinopharm Chemical Reagent Co., Ltd. Indium chloride tetrahydrate ($InCl_3 \cdot 4H_2O$, 99%), thioacetamide (CH_3CSNH_2, 98%) and copper nitrate ($Cu(NO_3)_2$, 98%) were obtained from Shanghai Maclean Biochemical Technology Co., Ltd.

2. Materials characterization

The crystal structure and composition of all samples was analyzed using an X-ray diffractometer (XRD, model Rigaku D/MAX gB, with Cu-Kα radiation, λ=0.1538 nm). The scanning angle 2θ ranged from 5° to 80° at a scanning speed of 5°/min. The elemental states and chemical composition of the samples were analyzed by the X-ray photoelectron spectroscopy (XPS, model PHI 5000C). The optical absorption capacity of the samples was characterized using a UV-visible diffuse reflectance spectrophotometer (UV-2600, Shimadzu, Japan). The bandgap width of the samples was obtained through data analysis using the Kubelka-Munk function. The morphology and elemental distribution of the samples were characterized using scanning electron microscopy (SEM, FEL NovaNano450), transmission electron microscopy (TEM, JEOL JEM-2100) and high-resolution TEM (HRTEM). Electron paramagnetic resonance (EPR) spectra were recorded to analyze the key reactive intermediate on a ER200-SRC, A300-10/12 spectrometer. The photoelectrochemical measurements were conducted using a CHI 660C workstation.

Formula S1

$$\text{Selectivity (\%)} = \frac{\text{moles of desired product}}{\text{moles of all products}} \times 100$$

Formula S2

$$\text{Overall carbon yield (\%)} = \frac{\text{Overall carbon of all products}}{\text{Overall carbon of consumed reactants}} \times 100$$

Formula S3

$$\text{Conversion (\%)} = \frac{\text{moles of consumed reactants}}{\text{moles of reactants}} \times 100$$

Formula S4

$$\alpha h\nu = A(hn - Eg)^{n/2}$$

Eg, α, h, ν and A denote the bandgap, optical absorption coefficient, Plank's constant, photonic frequency and a proportionality constant, respectively.

Formula S5

$$E_{fb}(\text{vs. NHE}) = E_{Ag/AgCl} + 0.059 \times pH + E^0_{Ag/AgCl}$$

Where $E^0_{Ag/AgCl}$ is 0.197 V, 0.5 mol/L Na_2SO_4 solution pH=6.9

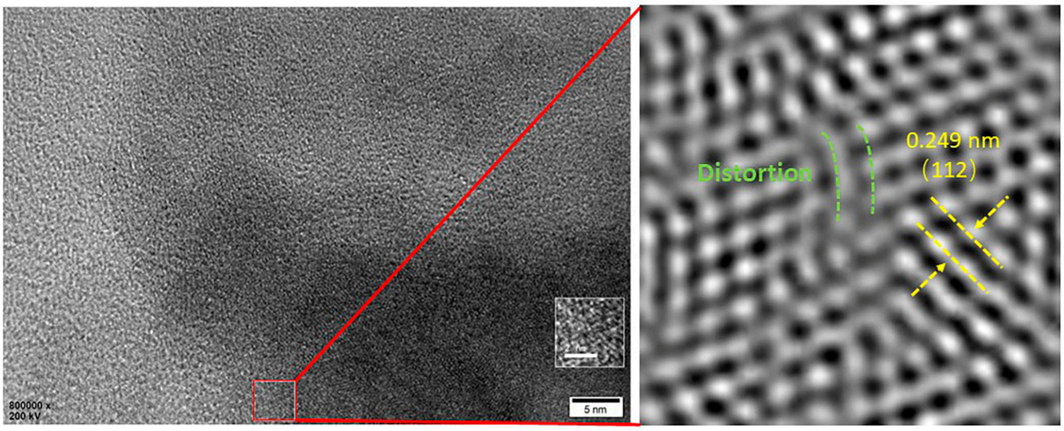

Figure S1. HRTEM images of $2Cu_3P/Cu_{0.5}ZIS$.

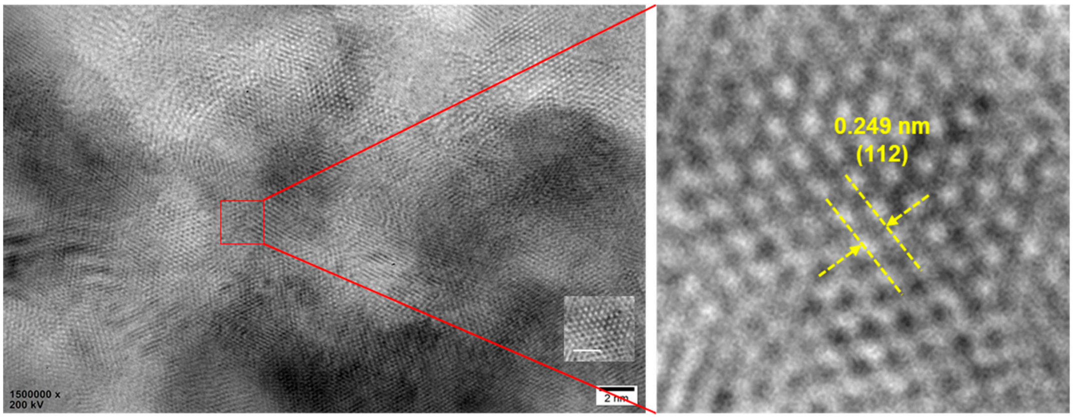

Figure S2. HRTEM images of $2Cu_3P/ZIS$.

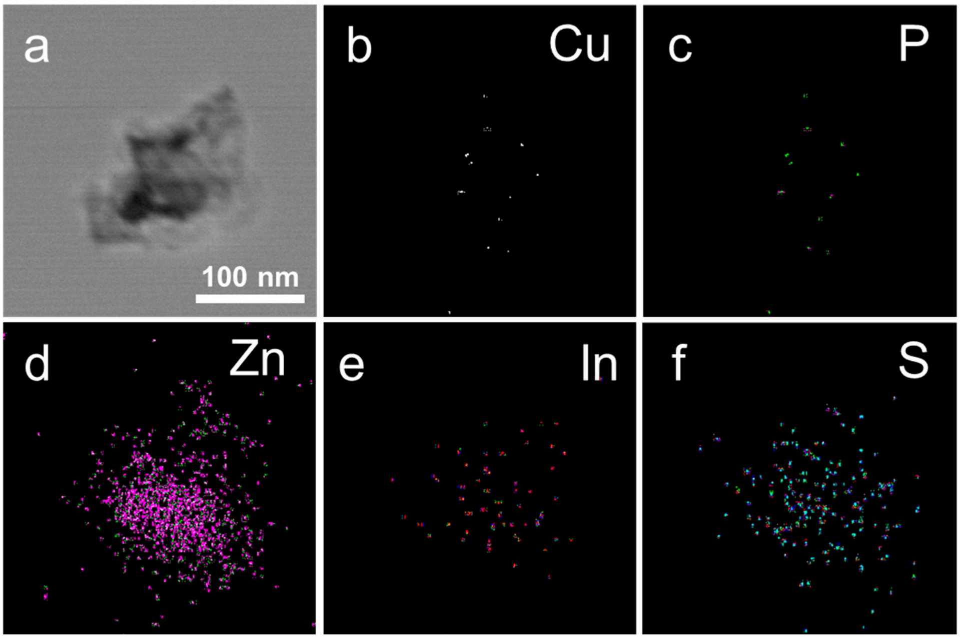

Figure S3. TEM (a) and the corresponding EDX element mapping images (b-f) of $2Cu_3P/Cu_{0.5}ZIS$ sample.

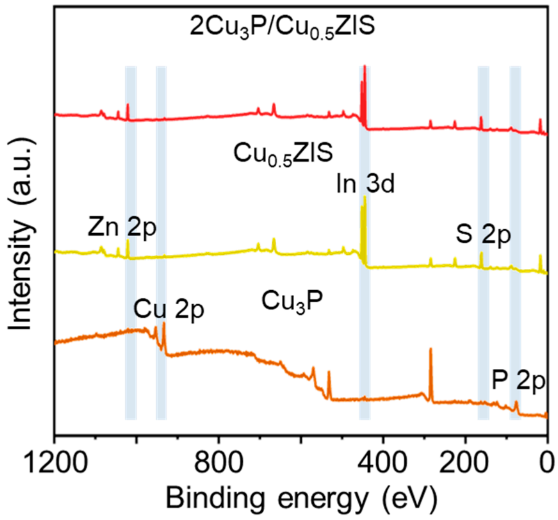

Figure S4. XPS survey spectra of Cu_3P, $Cu_{0.5}ZIS$ and $2Cu_3P/Cu_{0.5}ZIS$ samples.

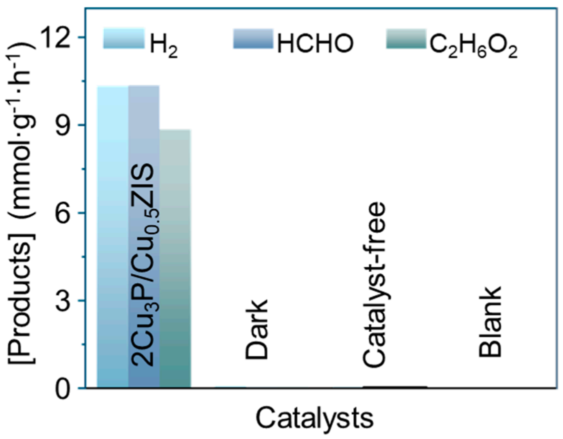

Figure S5. Control experiments for methanol conversion under different reaction conditions.

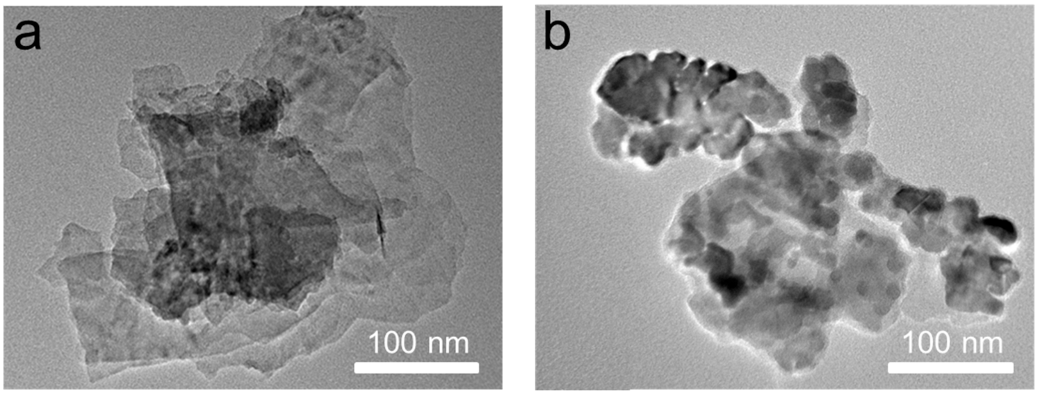

Figure S6. TEM images of $2Cu_3P/Cu_{0.5}ZIS$ sample before (a) and after (b) the reactions.

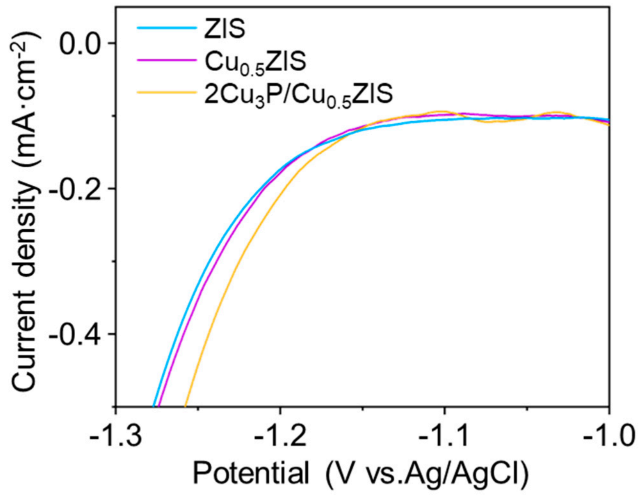

Figure S7. LSV curves of ZIS, $Cu_{0.5}ZIS$ and $2Cu_3P$/ $Cu_{0.5}ZIS$ samples.

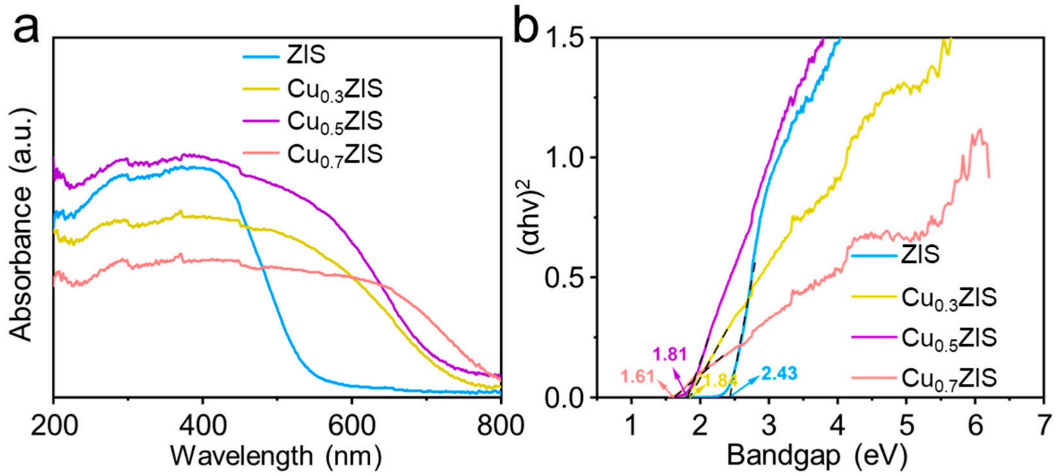

Figure S8. (a) DRS spectra of ZIS and Cu_xZIS (x=0.3, 0.5, 0.7) samples**.** (b) The band gap (Eg) value of ZIS and Cu_xZIS (x=0.3, 0.5, 0.7) samples.

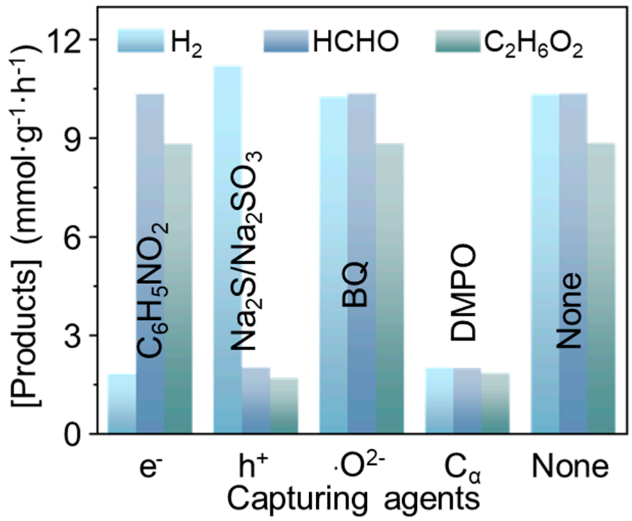

Figure S9. Free radical trapping experiment for methanol conversion in the presence of different scavengers.

Table S1. Methanol conversion performance of $2Cu_3P/Cu_{0.5}ZIS$

Catalyst	Formation rate ($mmol\cdot g^{-1}\cdot h^{-1}$)			Selectivity* (%)	
	HCHO	EG	HCOOH	HCHO	EG
$2Cu_3P/Cu_{0.5}ZIS$	10.35	8.84	0	53.93	46.07

* The selectivity was calculated on a molar carbon basis.

Table S2. ICP of Cu and P elements from $2Cu_3P/Cu_{0.5}ZIS$ after photocatalytic reaction.

Element	Concentration in the mixture ($\mu g\cdot mL^{-1}$)	Mass loss (%)
Cu	5.010	0.15
P	2.745	0.08
